# Isolation, Whole-Genome Sequencing, and Annotation of *Yimella* sp. RIT 621, a Strain That Produces Antibiotic Compounds against Escherichia coli ATCC 25922 and Bacillus subtilis BGSC 168

**DOI:** 10.1128/MRA.00329-19

**Published:** 2019-04-25

**Authors:** Anutthaman Parthasarathy, Narayan H. Wong, Nicolas D. Burns, Atlantis H. Aziz-Dickerson, Joyceline Dweh, D’Asia Buchanan, Michael A. Savka, André O. Hudson

**Affiliations:** a Thomas H. Gosnell School of Life Sciences, Rochester Institute of Technology, Rochester, New York, USA; b Rochester Prep High School, Rochester, New York, USA; University of Maryland School of Medicine

## Abstract

Here, we report the isolation, identification, whole-genome sequencing, and annotation of the bacterium Yimella sp. strain RIT 621. Concentrated spent medium extract treated with ethyl acetate was found to produce bactericidal compounds against the Gram-positive bacterium Bacillus subtilis BGSC 168 and the Gram-negative bacterium Escherichia coli ATCC 25922.

## ANNOUNCEMENT

Yimella is a genus of nonmotile coccoid Gram-positive bacteria belonging to the *Dermacoccaceae*, a family in the order *Actinobacteria* (1). The current literature shows that there have been only a few studies on this genus, most of which pertain to systematics (2, 3). As such, the biochemical pathways and the ecological roles of this genus have not been investigated and/or elucidated. One species, Yimella radicis, is an endophyte of the plant Paris polyphylla, and another species, Yimella lutea, is known to be halotolerant (2, 3).

**FIG 1:**
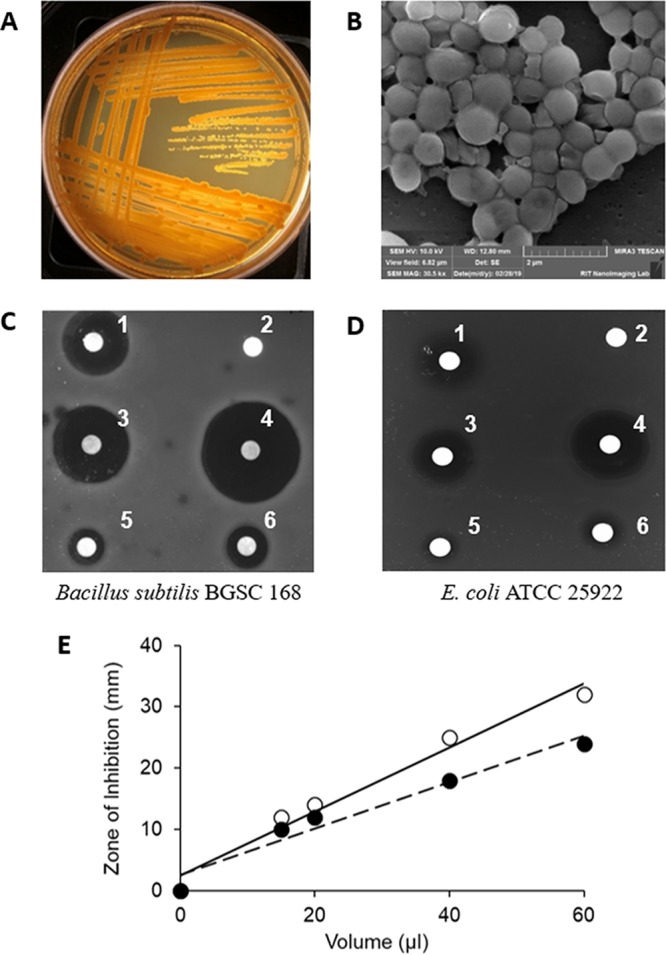
(A) Isolation of *Yimella* sp. strain RIT 621 streaked on tryptic soy agar. (B) Scanning electron micrograph (SEM) showing coccoid cells 0.5 to 0.8 µm in diameter (×30,500 magnification). (C) Susceptibility disk diffusion assay of *Yimella* sp. RIT 621 spent tryptic soy medium extract treated with ethyl acetate (500×) tested against Bacillus subtilis BGSC 168. (D) Susceptibility disk diffusion assay of *Yimella* sp. RIT 621 spent tryptic soy agar medium extract treated with ethyl acetate (500×) tested against E. coli ATCC 25922. 1, 20 µl tetracycline (10 mg/ml); 2, 20 µl methanol; and 3 to 6, 40 µl, 60 µl, 15 µl, and 20 µl *Yimella* sp. RIT 621 extract, respectively. (E) Graph showing the linear trend of the increase in diameter of the zone of inhibition (ZOI) versus increasing concentration of *Yimella* sp. RIT 621 extract.

Yimella sp. strain RIT 621 was isolated from the swab of a door handle located on the campus of the Rochester Institute of Technology by cultivation on tryptic soy agar at 30°C under aerobic conditions (Fig. 1A). The strain forms yellowish colonies on agar and, upon electron microscopy examination, shows clumps of coccoid cells about 0.5 to 0.8 µm in diameter joined by small projections (Fig. 1B). Genomic DNA was isolated from a 5-ml culture grown in tryptic soy broth using the PureLink microbiome DNA purification kit (ThermoFisher, USA), according to the manufacturer’s protocol. The bacterium was initially identified using PCR amplification and nucleotide sequencing of the variable (V3-V4) regions of the 16S rRNA gene using the following primers: 5′-CCTACGGGNGGCWGCAG-3′ and 5′-GACTACHVGGGTATCTAATCC-3′ (4). Taxonomic assignment of Yimella sp. RIT 621 was performed using the SILVA Alignment, Classification and Tree Service (ACT) tool (5). The FASTA 16S rRNA sequence was assigned to the family *Dermacoccaceae,* with a maximum identity of 95.72% to the SEED alignment. To obtain finer taxonomic resolution, the same FASTA 16S rRNA sequence was classified by a naive Bayesian model using the Ribosomal Database Project (RDP) Classifier tool (version 2.11) (6). RDP’s Classifier tool assigned the 16S rRNA sequence of RIT 621 to the genus Yimella with a minimum confidence threshold of 80%. For whole-genome sequencing, the genomic DNA was quantified using a Qubit 3.0 fluorometer, and the genomic DNA was processed using the Nextera XT library preparation kit (Illumina) for sequencing using the Illumina MiSeq platform at the Rochester Institute of Technology Genomics Facility. Libraries were sequenced using the MiSeq reagent kit version 3 for 2 × 150 cycles. Adapter trimming was done using the MiSeq Reporter software using the default parameters (sequences with 90% sequence identity to adapter sequences were trimmed). The trimmed FASTQ sequences were deposited into the Sequence Read Archive under accession PRJNA517609. The trimmed reads were subsequently assembled *de novo* with Unicycler version 0.3.0b (7). The genome annotation features are as follows: total assembly length, 3,271,355 bp; number of contigs, 48; GC content, 67%; total number of reads, 1,053,471; total number of bases sequenced, 316,041,300; coverage, 97×; number of open reading frames, 3,123; number of tRNAs, 47; and number of rRNAs, 4. The annotations are based on the National Center for Biotechnology Information (NCBI) Prokaryotic Genome Annotation Pipeline (PGAP) tool (8, 9).

Genome mining for antibacterial compounds using the antibiotics and Secondary Metabolite Analysis SHell (antiSMASH 4.0) Web server provided evidence that the bacterium possesses four gene clusters potentially encoding pathways for the synthesis of secondary metabolites, including terpenes, thiopeptides, and bacteriocin-like molecules (10). The antiSMASH analysis was supported by bactericidal activity using disk diffusion inhibitory assays against Bacillus subtilis BGSC 168 and Escherichia coli ATCC 25922 with ethyl acetate-treated spent extract from *Yimella* sp. RIT 621 (Fig. 1C and D). The bactericidal activity is slightly higher for *B subtilis* BGSC 168 than for E. coli ATCC 25922, as seen in the dosage plot comparing the zones of inhibition (ZOI) with increasing volumes of *Yimella* sp. RIT 621 extract (Fig. 1E).

### Data availability.

This whole-genome project for *Yimella* sp. RIT 621 has been deposited in GenBank under the accession number SEIP00000000. The version described in this paper is version SEIP01000000. The BioProject number is PRJNA517609, and the Biosample number is SAMN10839102.

## References

[1] Tille P 2015 Bailey & Scott’s diagnostic microbiology. Elsevier Health Sciences, St. Louis, MO.

[2] Yang LL, Jiang Z, Tang SK, Chu X, Xu LH, Zhi XY 2016 *Yimella radicis* sp. nov., an endophytic actinobacterium isolated from the root of *Paris polyphylla* Smith var. *yunnanensis*. Int J Syst Evol Microbiol 66:4191–4196. doi:10.1099/ijsem.0.001334. 27469463

[3] Tang SK, Wu JY, Wang Y, Schumann P, Li WJ 2010 *Yimella lutea* gen. nov., sp. nov., a novel actinobacterium of the family *Dermacoccaceae*. Int J Syst Evol Microbiol 60:659–663. doi:10.1099/ijs.0.013920-0. 19656924

[4] Herlemann DP, Labrenz M, Jürgens K, Bertilsson S, Waniek JJ, Andersson AF 2011 Transitions in bacterial communities along the 2000 km salinity gradient of the Baltic Sea. ISME J 5:1571–1579. doi:10.1038/ismej.2011.41. 21472016 PMC3176514

[5] Yilmaz P, Parfrey LW, Yarza P, Gerken J, Pruesse E, Quast C, Schweer T, Peplies J, Ludwig W, Glöckner FO 2014 The SILVA and “All-species Living Tree Project (LTP)” taxonomic frameworks. Nucleic Acids Res 42:D643–D648. doi:10.1093/nar/gkt1209. 24293649 PMC3965112

[6] Wang Q, Garrity GM, Tiedje JM, Cole JR 2007 Naïve Bayesian classifier for rapid assignment of rRNA sequences into the new bacterial taxonomy. Appl Environ Microbiol 73:5261–5267. doi:10.1128/AEM.00062-07. 17586664 PMC1950982

[7] Wick RR, Judd LM, Gorrie CL, Holt KE 2017 Unicycler: resolving bacterial genome assemblies from short and long sequencing reads. PLoS Comput Biol 13:e1005595. doi:10.1371/journal.pcbi.1005595. 28594827 PMC5481147

[8] Tatusova T, DiCuccio M, Badretdin A, Chetvernin V, Nawrocki EP, Zaslavsky L, Lomsadze A, Pruitt KD, Borodovsky M, Ostell J 2016 NCBI Prokaryotic Genome Annotation Pipeline. Nucleic Acids Res 44:6614–6624. doi:10.1093/nar/gkw569. 27342282 PMC5001611

[9] Haft DH, DiCuccio M, Badretdin A, Brover V, Chetvernin V, O’Neill K, Li W, Chitsaz F, Derbyshire MK, Gonzales NR, Gwadz M, Lu F, Marchler GH, Song JS, Thanki N, Yamashita RA, Zheng C, Thibaud-Nissen F, Geer LY, Marchler-Bauer A, Pruitt KD 2018 RefSeq: an update on prokaryotic genome annotation and curation. Nucleic Acids Res 46:D851–D860. doi:10.1093/nar/gkx1068. 29112715 PMC5753331

[10] Blin K, Wolf T, Chevrette MG, Lu X, Schwalen CJ, Kautsar SA, Suarez Duran HG, de Los Santos ELC, Kim HU, Nave M, Dickschat JS, Mitchell DA, Shelest E, Breitling R, Takano E, Lee SY, Weber T, Medema MH 2017 antiSMASH 4.0—improvements in chemistry prediction and gene cluster boundary identification. Nucleic Acids Res 45:W36–W41. doi:10.1093/nar/gkx319. 28460038 PMC5570095

